# Social Jetlag and Related Risks for Human Health: A Timely Review

**DOI:** 10.3390/nu13124543

**Published:** 2021-12-18

**Authors:** Rocco Caliandro, Astrid A. Streng, Linda W. M. van Kerkhof, Gijsbertus T. J. van der Horst, Inês Chaves

**Affiliations:** 1 Department of Molecular Genetics, Erasmus MC Cancer Institute, Erasmus University Medical Centre Rotterdam, 3015 GD Rotterdam, The Netherlands; r.caliandro@amsterdamumc.nl (R.C.); a.streng@erasmusmc.nl (A.A.S.); g.vanderhorst@erasmusmc.nl (G.T.J.v.d.H.); 2 Centre for Health Protection, National Institute for Public Health and the Environment (RIVM), 3721 MA Bilthoven, The Netherlands; linda.van.kerkhof@rivm.nl

**Keywords:** social jetlag, chronotype, circadian misalignment, sleep, health risk

## Abstract

The term social jetlag is used to describe the discrepancy between biological time, determined by our internal body clock, and social times, mainly dictated by social obligations such as school or work. In industrialized countries, two-thirds of the studying/working population experiences social jetlag, often for several years. Described for the first time in 2006, a considerable effort has been put into understanding the effects of social jetlag on human physiopathology, yet our understanding of this phenomenon is still very limited. Due to its high prevalence, social jetlag is becoming a primary concern for public health. This review summarizes current knowledge regarding social jetlag, social jetlag associated behavior (e.g., unhealthy eating patterns) and related risks for human health.

## 1. Introduction

Like most forms of life, humans and other mammals have an internal body clock with a self-sustained, near 24 h periodicity, referred to as the circadian clock (from the Latin circa diem, meaning about a day). This system confers rhythmicity to a variety of behavioral and physiological processes (e.g., sleep, body temperature, heartbeat rate, metabolism, hormone secretion) and neurobehavioral processes and allows an organism to anticipate daily recurring environmental changes, such as the light-dark cycle, food availability, and predator activity. To remain synchronized with the day-night cycle, the circadian clock is daily reset by light, the primary entraining signal for circadian rhythms [1,2].

Over the last 200 years, the human lifestyle has dramatically changed as a result of the modernization of our society, the widespread availability of artificial light, the nightwork inherent to our 24/7 economy, and the possibility of rapid travelling across time zones [3,4]. While these technological improvements have undoubtfully eased our daily life (e.g., constant access to light, energy, and food), they also introduced a new phenomenon in our population, known as “circadian misalignment” [3,4,5]. Circadian misalignment occurs when there is a mismatch between the environmental time and the body’s internal time and is typically observed in people who experience jetlag or in shift workers [6,7]. Given the wide range of physiological processes regulated by the circadian clock, circadian misalignment is thought to negatively impact human health, with effects in the short- and long-term [3,5]. It has been proposed that circadian misalignment is linked to acute effects such as poor and shortened sleep, impaired alertness, poor performance, hypertension, and abnormal inflammatory status [8,9,10,11]. In the long-term, circadian misalignment is associated with a higher risk to develop obesity, diabetes mellitus, metabolic syndrome (MetS), cancer, cardiovascular diseases, cognitive impairments, and Alzheimer’s disease [1,7,12,13,14,15,16].

Whereas the adverse health effects of shift work and jetlag have been widely recognized, there is another, less evident and accordingly highly underestimated, form of circadian misalignment known as “social jetlag” (SJL) [17,18,19]. A major trigger for developing SJL are school, work and other social obligations, forcing people to use the alarm clock to start the day ahead of their natural wake up time. Additionally, SJL can occur when people have to stay awake beyond their natural bedtime. In industrialized countries, the majority (70%) of the population is faced with a SJL ranging from 1 h to over 2 h [17,18,19].

This review summarizes current knowledge on SJL and its effect on human health, highlights the current gaps of knowledge, and suggests new strategies to improve and expand our current understanding of this phenomenon.

## 2. The Mammalian Timekeeping System

### 2.1. The Mammalian Circadian Clock

The mammalian circadian system is organized in a hierarchical manner, with a master clock located in the suprachiasmatic nucleus (SCN) of the hypothalamus, and peripheral clocks in virtually all other cells and tissues of the body [1,20,21]. To keep pace with the exact 24 h day-night cycle, the circadian system requires daily resetting by environmental time ques, known as Zeitgebers (time cue; from the German words zeit = time, and geber = giver) [1,21,22]. As light is the most robust and predictable environmental Zeitgeber, it may not come as a surprise that the master clock is daily reset by light in a process called photoentrainment [1,23]. Photic information is processed by a specialized class of retinal photoreceptors, called intrinsically photosensitive retinal ganglion cells (ipRGCs) [20,23]. Morning sunlight, typically characterized by short wavelengths (i.e., blue light of ~480 nm), triggers the strong activation of the ipRGCs while the evening sunlight—characterized by longer wavelengths (>600 nm)—only has a minimal effect on those cells [20,23]. The photic information is received by the ipRGCs and transmitted to the SCN via the retinohypothalamic tract. Signals from the SCN synchronize clocks of peripheral cells and tissues through various neural and humoral signals [1,20,21,24].

At organ and tissue level, peripheral oscillators promote circadian rhythmicity of tissue-specific physiological processes [21,22,25]. In addition to the signals incoming from the SCN, peripheral oscillators can be entrained by non-photic Zeitgebers such as stress, physical exercise, hypoxia, and food intake [26,27,28]. For example, exposure of mice to forced activity, time-restricted feeding (TRF; i.e., 12–20 h fasting) or other stressful stimuli under dim light conditions has been shown to phase shift the clock gene expression profile in the liver [25,27]. The expression of peripheral clock genes in the liver, kidney and brain areas, except for the SCN, is changed already by one night of fasting in mice [29]. Additionally, it was shown that eating during the inactive period results in internal desynchronization at different levels [30]. These studies indicate that feeding cues are more dominant than light cues for peripheral clock adjustment. However, a recent study on the impact of more natural (i.e., timed) feeding patterns (as compared to ad libitum food intake) revealed that the magnitude of the phase shift registered in peripheral tissue is limited and smaller than originally thought, indicating that an unnatural TRF regime rather than the feeding behaviors entrain the peripheral tissues [31]. However, it should be noted that the TRF time was 12 h and TRF was not investigated in the dark (active) phase. In general, non-photic Zeitgebers, such as timing of food intake, may be able to cause entrainment of the peripheral tissues, in addition to photic-induced entrainment via the SCN [24,25,27,31].

### 2.2. Molecular Basis of the Mammalian Circadian Clock

Circadian rhythms are generated by a self-sustaining, cell-autonomous molecular oscillator composed of a set of clock genes and proteins that act in so-called transcription-translation feedback loops [32,33]. In mammals, the transcription activators *Brain and Muscle ARNT-Like1* (*BMAL1*) and *Circadian Locomotor Output Cycles Kaput* (*CLOCK*) form a heterodimer (CLOCK/BMAL1 complex) which activates the transcription of the *PERIOD (PER)* and *CRYPTOCHROME (CRY)* genes through E-box elements in their promoter [33,34]. The resulting PER and CRY proteins translocate to the nucleus where they form a heterodimer (PER:CRY complex) that inhibits the activity of CLOCK:BMAL1 complex, ultimately resulting in the downregulation of *CRY* and *PER* gene expression (negative feedback loop) [35,36,37]. Degradation of PER:CRY complex allows again the start of a new cycle [33,34]. In addition to regulating their own cyclic expression, core clock genes drive rhythmic expression of E-box containing clock-controlled output genes, including several transcriptional activators and inhibitors. Due to this cascading effect, over 20% of a tissue transcriptome is cyclically expressed, with mRNA levels peaking at defined stages throughout the circadian cycle [33,34].

Studies with cultured fibroblasts from short period *Cry1* and long period *Cry2* mutant mice have shown that the genetic makeup of peripheral clocks is similar to that of the SCN clock and that the periodicity of cellular oscillations reflects that of the free-running behavioral rhythm [38]. Likewise, this in vitro approach was used to demonstrate that the period length of the mammalian clock varies among people [33,39]. To a large extent, this interindividual variability of periodicity is due to differences in the protein stability of the core-clock gene products resulting from post-translational modifications [33].

### 2.3. Chronotype

Chronotype is the term used to describe a person’s innate preference for timing of sleep and activity, as exemplified by the existence of morning and evening people (also known as larks and owls), and to a large extent reflects the underlying phase of entrainment [18,40]. In the human population, chronotypes exhibit a near-normal distribution, with most chronotypes being intermediate between the extremely morning types, of those who have a biological predisposition to be active very early in the morning, and the extremely evening types, typical of those who have the tendency to be active very late in the evening [18]. Chronotype reflects the timing of peak cognitive and physical performance: morning types have their peak cognitive performance in the morning and their peak physical performance in the afternoon, while evening types have their peak performances in the late afternoon/evening or, in the most extreme cases, even at night [41]. To classify chronotype, Roenneberg and colleagues have developed the Munich Chronotype Questionnaire, which examines a person’s sleep behavior and allows computation and scaling of chronotype on the basis of local time of midsleep (the midpoint between sleep onset and wake up) on free days [18]. Using the MCTQ, human chronotype has been shown to exhibit a near-normal distribution, ranging from (extremely) early to late, with the majority of individuals having an intermediate chronotype being intermediate [18]. Interestingly, in vitro studies have shown that the period length of the molecular oscillator of cultured fibroblasts correlates with sleep preference and chronotype [38,40], indicating that clock period is an important determinant of chronotype.

Importantly, intrinsic factors such as gender and age are known to modulate chronotype, as shown by MCTQ-based population studies [42,43]. In general, children tend to be early types, with no evident differences between males and females [42,43]. However, starting at puberty, chronotype has been shown to drift toward eveningness, peaking at late adolescence and usually more pronounced in men than in women. In general, during adulthood chronotypes progressively shift back toward morningness with increasing age [42,43,44]. Inherent to their innate late bed and wake up time, subjects with a late chronotype tend to be more out of sync with social norms than other types [45,46].

Last but not least, as chronotype is expressed in local time, it should be noted that the geographical position of an individual’s place of residence has an impact on the chronotype. For example, after averaging chronotype data from the German population in 9 longitudinal bins spanning one degree, Roenneberg and colleagues observed a 34 min increase in chronotype from east (15° E) to west (9° E) [47]. This leads to the conclusion that the circadian clock follows solar time (e.g., sunrise moving 4 min per longitudinal degree) rather than social time (as chosen for a time zone and only corresponding to solar time at the meridian) [47,48].

## 3. Social Jetlag

The term social jetlag (SJL) refers to a form of circadian misalignment that arises from the discrepancy between activity/sleep schedules on school/work days and free days [17]. In the studying/working population of industrialized countries, SJL is a highly prevalent phenomenon that many people experience for the whole study/work career [6,17,49]. It has been estimated that 70% of students and workers experience at least one hour of SJL, while almost half of them experience two hours or more [17,19]. Additionally, SJL is positively associated with sleep deficit, which makes it disputable whether the effects of SJL are due to circadian misalignment or sleep loss [50]. Most of the time, people cannot personalize the schedules of school or work, instead, they need to follow a standardized (often) early schedule (e.g., a nine-to-five job) that does not take into account individual preferences. Overall, on free days, people tend to have an activity pattern that better reflects their circadian preferences [17]. Considering the high percentage of the population at risk of SJL and associated sleep loss, this form of circadian misalignment is now a prior concern for public health.

To determine the amount of SJL that a person experiences, it is necessary to compare the phase of entrainment on school/work days and free days [17]. The phase of entrainment can be assessed subjectively using questionnaires such as the Munich Chronotype Questionnaire (MCTQ) [17,18]. Alternatively, measuring circadian “phase markers” such as dim-light melatonin onset (DLMO) or core body temperature (CBT) [51] may represent an objective way to determine the phase of entrainment. However, external factors such as environmental light exposure, carbohydrate intake, or excessive physical activity, are known to influence these proxy markers for circadian phase, making their use in determining social jetlag more challenging [52,53]. Moreover, measuring DLMO would require multi-sampling over the 24 h which, in the context of medium and large-scale human cohort studies, is expensive and has a larger impact on the daily routine of participants [54]. Hence, the majority of epidemiological studies on chronotype is based on subjective questionnaires rather than measurement of objective proxy markers [55].

### 3.1. Assessment of Social Jetlag

**Figure 1:**
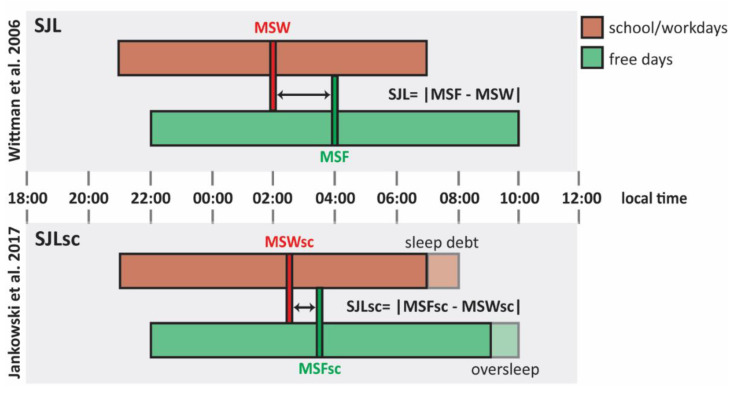
Social jetlag (SJL) and sleep debt-corrected SJL (SJL_SC_). Comparison between social jetlag (SJL) calculated as proposed by Wittman et al. [17] and sleep debt-corrected SJL (SJL_SC_) as proposed by Jankowski et al. [58]. This schematic representation shows the hypothetical sleep episodes of a late chronotype with an early schedule from Monday to Friday. Bars illustrate sleep episodes, as well as their timing (expressed in local time) and duration, on school/work days (red bars) and free days (green bars). Following the formula proposed by Jankowski et al. [58] (lower part of the illustration), MSW_SC_ results delayed if compared to MSW due to sleep debt school/work days (red dashed block), while MSF_SC_ is anticipated if compared to MSF because of the oversleep of free days (green dashed block). Abbreviations: MSF, midsleep on free days; MSW, midsleep on work days; MSF_SC_, oversleep-corrected midsleep on free days; MSW_SC_, sleep debt-corrected midsleep on work days.

Initially, Wittmann and colleagues mathematically defined SJL as the absolute difference between the midsleep point on free days (MSF) and the midsleep point on work (or school) days (MSW), both expressed in local time  (SJL=|MSF - MSW|) [17]. However, during work days, many people sleep less than their genetically determined sleep need, thereby accumulating a sleep debt that is counterbalanced with oversleeping on free days [18,42]. Since oversleep on free days delays MSF and, oppositely, sleep loss on work days advances MSW, ignoring this situation will lead to an overestimation of a person’s SJL [56,57]. Although a midweek sleep debt may not always be fully compensated for on weekend days, it has been proposed that the impact of sleep loss on SJL estimation can be minimized using a sleep corrected version of MSF and MSW [57,58]. MSF_SC_ and MSW_SC_ should be calculated as the sum of average sleep duration across the whole week and sleep onset on free days (SO_F_) and work days (SO_W_), respectively (MSF_SC_ = SO_F_ + ½ SD_week_; MSW_SC_ = SO_W_ + ½ SD_week_) [57,58]. The resultant sleep corrected midsleep point on free days (MSF_SC_) and sleep corrected midsleep point on school/work days (MSW_SC_) can be used to quantify the sleep corrected SJL ${(\mathrm{SJL}}_{\mathrm{SC}}{=|\mathrm{MSF}}_{\mathrm{SC}\;}{-\;\mathrm{MSW}}_{\mathrm{SC}}\left|\right)$ [57,58]. It should however be noted that SJL_SC_ actually corresponds to the difference in sleep onset time on free and work days and that the question remains open to what extend MSF or MSF_SC_ based SJL calculations correlate with adverse health risk (Figure 1) [57,58].

### 3.2. Factors Affecting the Incidence and Magnitude of Social Jetlag

Many sociodemographic and behavioral factors have been reported to modulate SJL [59]. Similar to the relationship between chronotype and geographical location, showing a westbound gradient from early to late chronotype [47], few studies showed that the incidence of SJL is increased in people who live close to the west boundary of a time zone [59,60]. Importantly, the risk of developing SJL will further increase in situations where the standard time in a time zone (i.e., social clock) further deviates from solar time. In some cases, this discrepancy can be extremely pronounced, as exemplified by the western part of China, where the standard time is set according to Beijing time and accordingly deviates 6 h from local solar time [59].

Daylight saving time (DST), the practice of setting the clock time one hour forward in spring and returning to standard time in autumn, even generates a further discrepancy between social and solar time [59]. During daylight saving time, people are forced to advance their daily routine, with earlier wake-up and early bedtimes. The impact of DST is best illustrated by a recent study on SJL in the Russian population before and after the 2011 switch from daylight saving time (biannual clock change) to permanent daylight saving time, and subsequent switch to permanent standard time in 2014 [48]. Permanent daylight-saving time was shown to cause an increase in the incidence and magnitude of SJL, when compared to permanent standard time [48,59].

Another factor that has been reported to correlate with SJL is the use of light emitting electronic devices before bedtime. Studies conducted both in young adults and adolescents have shown that the use of computers or phones a few hours before going to bed was associated with a stronger SJL [61,62,63]. The blue light coming from computer and phone screens interferes with circadian rhythms, especially when exposure occurs in the evening [63].

Whereas studies specifically addressing gender-specific differences in SJL are currently lacking, circadian misalignment has been proposed to have different effects on males and females. In 2016, Santhi and colleagues used a desynchrony protocol to examine the effects of the circadian misalignment in 16 men and 18 women and showed gender-specific quantitative differences in sleepiness, mood alteration, and cognitive impairment [64]. In 2019, Qian and colleagues showed how circadian misalignment differently impairs the regulation of energy homeostasis in males and females [65]. This study showed that males experience increased hedonic appetite (consumption of food for pleasure) with no changes in energy expenditure, while females manifest more disturbances of the energy homeostasis processes [65]. Moreover, as males on average have a later chronotype and as late chronotype is associated with higher risk to experience SJL [42], it would be legit to think that the incidence, magnitude and/or effects of SJL-related physical and mental complaints are subject to gender specific differences. Further studies will be required to investigate the sex-specific differences in SJL.

Interestingly, lockdown measures during the global COVID-19 pandemic forced the majority of people to work from home, thereby avoiding commuting traffic and being more flexible in working hours. Generally, a survey of the sleep characteristics of people before and during the COVID-19 pandemic revealed that sleep duration on weekdays became longer, and sleep onset and sleep offset were delayed and these parameters were more comparable to the sleep duration of the free days, resulting in a decreased SJL [66,67,68]. In addition to a one hour reduction in circadian misalignment, Korman and colleagues noticed a substantial drop of 70% in the use of the alarm clock to wake up, especially in evening types, pointing to the alarm clock as a predisposing condition for SJL [66].

## 4. Adverse Effects of Social Jetlag

The necessity to maintain more regular sleep/activity patterns comes from epidemiological and animal studies identifying jetlag and shiftwork as risk factors for a variety of disorders, including obesity, diabetes, and cancer [20]. Although SJL is a less stringent form of circadian misalignment than aforementioned conditions, there is increasing evidence that SJL may also be predisposing for several pathological conditions. These will be discussed in the next paragraph.

### 4.1. Impaired Sleep and Cognitive Performance

Wittman and colleagues were the first to observe that the sleep of people experiencing SJL, particularly of the evening types, tends to be short and poor during school/work days [17,19]. Particularly, SJL can generate a sleep debt or even chronic sleep deprivation, poor sleep quality and cognitive performance [17,19].

A recent small cohort study conducted on 33 healthy young male students, investigated the association between SJL and poor sleep during school/work days [69]. Sűdy and colleagues examined the intra-individual changes in heart rate variability (HRV) as indicator of sleep quality, in people with high or low SJL between work day and free days. In the first 3 h of sleep the group with high SJL had a lower HRV on a work day, as compared to a free day, while the HRV values of the low SJL group remain constant between the work and free day. Based on these data, SJL seems to be correlated with a lower quality of sleep [69]. The effects of SJL on sleep quality have also been investigated in shift workers. Due to their working schedule, all shift workers have to periodically re-adjust their wake-up and bedtimes to be able to attend morning, evening or night shifts. As these shifts are usually assigned regardless of the individual’s chronotype, it is no surprise that shift workers can experience up to three hours of SJL [70,71]. Previous studies revealed that shift workers that experience SJL are also characterized by short sleep duration and low sleep quality [70,71]. In case of poor quality, sleep might exert a lower restorative capacity that could explain why people who experience SJL tend to be less alert, more fatigued, less prompt to wake up in the morning, and perform worse in academia or the workplace than those who do not experience SJL [72,73,74,75,76].

In addition to the potentially lower quality of sleep, people who experience SJL are often sleep-deprived [42,43]. This is particularly common in evening types as they, on the one hand, cannot fall asleep early in the evening and, on the other hand, have to wake up early in the morning, due to social obligations [42,43]. As a result, the sleep of the evening types is shortened from both sides, resulting in significant sleep deprivation during school/work days.

### 4.2. Metabolic Diseases

#### 4.2.1. Metabolic Changes

To shed light on the impact of SJL on metabolism, previous studies have investigated a variety of metabolic risk blood markers in people who experienced SJL. A study among 145 healthy adults (54% females) revealed that a SJL of more than 2 h associated with increased (fasting) cortisol levels [49]. Using a similar approach, Wong and colleagues investigated a group of 447 middle-aged healthy adults (53% females) and observed that SJL associates with low levels of high-density lipoprotein (HDL) cholesterol, and high levels of triglycerides, which are metabolic risk factors [77]. A third study, with 792 middle-aged adults (73% females), showed that higher levels of total cholesterol, triglycerides and fasting glucose are associated with SJL [78]. These findings suggest that SJL is linked to metabolic changes that may in turn increase the risk of MetS [77,78].

Metabolic changes can (in part) stem from unhealthy behaviors that people experience under SJL conditions. Although the previous studies have corrected for some lifestyle and behavioral covariates [49,77,78], there is a multitude of unhealthy behaviors that are recurrent in people experiencing SJL and that could be either confounders or mediating factors for metabolic changes. For example, in terms of dietary food intake, people experiencing SJL were found to consume more high-calorie and high-sugar foods (e.g., meat, eggs, sweets, soft drinks) and less healthy foods (e.g., fruits, vegetables) [50,79,80]. Additionally, individuals that experience SJL often show less adherence to healthy dietary patterns [81,82]. Of note, a recent study compared the dietary food intake of 710 young Japanese adults (72% females) on school/work days vs. free days. The mean energy, fat, saturated fat, and monounsaturated fatty acids intake of subjects undergoing SJL were significantly increased on free days, as compared to control subjects not experiencing SJL [83].

Interestingly, a combination of SJL and the so-called “cafeteria diet (CafD)”—enriched in highly caloric foods—was able to cause severe metabolic changes in rats, while SJL or CafD alone caused only mild metabolic changes [84]. These findings provide experimental evidence that the presence of both conditions lead to an increased risk of an adverse metabolic profile [84]. In line with this observation, Oosterman and colleagues [85] have shown that there is a combined effect of diet composition and timing on hepatic fat accumulation and disturbance of circadian clock gene expression in rats.

In addition to less healthy dietary patterns, other unhealthy behavioral characteristics of people with higher levels of SJL include stronger sleep deprivation, higher tobacco consumption, reduction of physical activity, longer eating duration, and later timing of food intake [76,79,84,86,87]. With respect to later dinner timing, it has been proposed that eating close to the DLMO may reduce the thermic effect of food, resulting in a more positive energy balance and thus weight gain [79,88].

#### 4.2.2. Obesity

A few studies have shown that SJL is associated with higher Body Mass Index (BMI) and higher waist circumference, both predictive markers for obesity [19,77,89,90]. In contrast, a Dutch study investigating SJL and obesity-related characteristics among Dutch teenagers did not confirm the association between SJL and BMI [91]. A few limitations might have influenced the results of this study, including the small cohort selected consisting of 83 Dutch teenagers with only 7% overweight subjects and 2% obese subjects [91]. It is important to notice that all the studies that confirmed the association between SJL and BMI or waist circumference calculated the SJL from the participants’ MCTQ without correcting for sleep debt bias. In a recent study SJL was measured in an objective manner, based on wrist temperature (T), physical activity (A), and body position (P) [92]. In this study, involving 432 healthy children an association between SJLSC, evening chronotype, and increased BMI was observed. According to the authors, the integrative measurement (TAP) may represent the ultimate methodology to study a person’s circadian phase, as it seems to be more reliable than the measurement of proxy markers and it is not very invasive [92,93]. Nevertheless, this approach will require further validation in future studies focused on SJL.

#### 4.2.3. Diabetes

Another metabolism-related disease that has been linked to SJL is diabetes. For example, based on the New Hoorn Study cohort (*n* = 1585, 53% females, mean age 60.8 ± 6.0 years), Koopman and colleagues conclude that 2 h of SJL associates with an approximately 2-fold increased risk of both pre-diabetes and type-2 diabetes (T2D) [94]. Another study with 447 middle-aged healthy adults (53% females) shows that SJL correlates with abnormal carbohydrate homeostasis, higher fasting plasmatic insulin concentrations, and higher insulin resistance [77]. A longitudinal study involving 625 individuals (76% females, mean age 56.0 ± 12.0 years) with non-communicable chronic diseases (NCCDs, including diabetes, systemic arterial hypertension, obesity, or dyslipidemia) reported a positive association between SJL and the fasting glucose levels in the overall cohort and in the T2D group [95]. In this study, the authors did not find an association between SJL and the glycated hemoglobin (HbA1c), diagnostic marker used to assess the glucose levels in the blood [95]. In contrast with these results, two independent type-1 diabetes (T1D) patient cohort studies revealed the association between SJL and poor glycaemic control, indicated by high HbA1c levels [86,96]. One of the main issues of these studies, however, is that SJL was not corrected for the sleep debt as proposed by Jankowski, which could have led to an overestimation of the SJL [58]. Furthermore, for some of these studies, SJL was derived from self-reported sleep times [78,96] or MCTQ of the participants [94], while only two studies determined SJL from the objective measure of sleep patterns [77,86]. Nevertheless, despite the limitations, these findings do suggest an association between SJL and diabetes.

### 4.3. Adverse Cardiovascular Outputs

Several studies have investigated the association between cardiovascular effects and SJL [49,97]. As mentioned in Section 4.1, Sűdy and colleagues compared the autonomic cardiac functionality (evaluated in terms of HRV) of subjects with high SJL and subjects with low SJL [69]. The authors detected, in the group with high SJL, an intra-individual reduction of the HRV on the school/work days and free days, indicating an increased risk of adverse cardiovascular outputs that could be associated with SJL [69,98]. In another cohort study of 145 healthy subjects (55% females), people who experienced more than 2 h of SJL were found to have significantly higher resting heart rate compared to those who experienced less than 1 h of SJL [49]. In line with this, an epidemiological study of shift workers found an association between SJL and altered heart rate [97]. However, Wong and colleagues could not confirm the association between SJL and altered heart rate [77]. This could be because Wong and colleagues calculated SJL from the objective measure of participant’s sleep/activity patterns by actigraphy by measuring for 7 days, which is too short to give reliable data on SJL [77]. In the last decade, four independent studies have investigated the relation between SJL and blood pressure [99]. Despite the heterogeneity of the studied cohorts, none of the studies found an association between these two phenomena [99].

In the previous studies, SJL was estimated from self-reported sleep schedules [49,97]. Furthermore, none of these studies corrected the SJL of the participants for the sleep debt, indicating that a potential bias could affect the results. Considering that heart rate is a predictor of cardiovascular mortality both in the general population and in patients with cardio- and cerebrovascular diseases, it is fundamental that future studies clarify whether an association with SJL exists [100].

### 4.4. Psychiatric Disorders

Epidemiological studies have linked eveningness to adverse mental outcomes such as emotional/behavioral problems, mood disorders, depression, and bipolar disorder [101,102,103,104]. To investigate the impact of SJL, Levandovski and colleagues conducted a study on 4051 healthy Brazilian subjects (67% females), and a positive correlation between depth of depressive symptomatology and hours of SJL [45]. Subjects who experienced 2 or more hours of SJL showed more severe depressive symptoms than those who were experiencing fewer hours of SJL. The main weakness of this study is that SJL was not sleep debt-corrected and therefore potentially biased [45]. Recently, however, Islam and colleagues conducted a cross-sectional study in Japanese non-shift workers, aiming to investigate the association between depressive symptomatology using the sleep-debt corrected SJLSC [105]. Their finding that SJLSC was associated with an increased likelihood of having depressive symptoms further supported the original findings of Levandovski and colleagues [45].

In 2018, Knaper and colleagues were the first to study the association between SJL and depression in a clinical sample (so far only studies on the general population were conducted). They showed that patients diagnosed with Major Depressive Disorder (MDD) do not experience more SJL than healthy controls do, although SJL was derived from self-reported questionnaires asking for sleep timing data of the weeks before the study [106].

The link between SJL and psychotic disorders other than depression, such as anxiety and attention deficit hyperactivity disorder (ADHD), has been investigated [107,108,109,110]. Polugrudov and colleagues carried out a study on a cohort of 62 young subjects (57% females) that highlighted no correlation between the severity of trait anxiety and the hours of SJL [107]. Similar negative results were seen in two other cohort studies [108,109]. Finally, in a recent study conducted on 492 young subjects (73% females), McGowan and colleagues determined that ADHD symptoms are associated with evening types and SJL [110].

Studies performed so far with the aim to identify an association between SJL and psychiatric or mood disorders have contradicting results. This could be a result of differences in study population, disorder, or methodology. It is therefore important that more studies with objective measures are performed to further analyze a role of SJL in the severity of mood disorders.

## 5. Conclusions and Future Perspectives

In this review, we discussed the impact of social jetlag on the well-being and health of humans. According to these studies, SJL seems to be associated with a lower academic and/or work performance during school/work days. Additionally, people who experience SJL might be at a higher risk to develop an adverse metabolic profile involving, among others, changes in cortisol regulation and dyslipidemia and metabolism-related disorders, such as obesity and T2D. Metabolic changes might result, at least in part, from unhealthy behaviors that are recurrent in people who experience SJL. Furthermore, some available studies indicate the existence of an association between these SJL and cardiovascular outputs, such as HRV and resting heart rate. With respect to the association between SJL and depression, conflicting results have been published. In conclusion, evidence is increasing that social jetlag, such as night work, is a health risk factor and predisposes to later life disease. Yet, our understanding of how chronic exposure to SJL ultimately translates into morbidity, as well as how this adverse effect SJL can be counteracted by intervention strategies is still limited and presents some considerable gaps.

One such gap is that SJL has been studied almost entirely through epidemiological studies. Whereas population studies are highly suitable to disclose associations, heterogeneity between individuals (including duration of chronic SJL exposure, genetic makeup of the molecular clock as reflected by chronotype), ethical reasons, and the time-consuming and costly nature of such studies often preclude establishment of causal relationships. Animal studies involving transgenic laboratory animals with well-defined chronotypes in an otherwise genetically homogeneous background provide a solution to this problem, as illustrated by Espitia-Bautista and colleagues [84]. Using a programmable rotating cage to force awakening a specific time point on week and weekend days, they provided experimental evidence that SJL in combination with carbohydrate and fat rich food predisposes to obesity and metabolic syndrome in rats [84]. This approach can be further improved by employing short-period *Cry1* and long-period *Cry2* knockout mice, which have been proposed as rodent models for morningness and eveningness, respectively [111,112]. Such animal models represent important tools to study SJL, as within lab settings researchers can control for confounders such light conditions and lifestyle factors.

Another gap is the absence of objective biomarkers to quantitatively assess the level of circadian misalignment including SJL. Furthermore, whereas currently used proxy markers such as melatonin and cortisol [113], but also the integrated TAP measurement (temperature, physical activity, and body position) [92,93], may be helpful to obtain a snapshot view (i.e., measured over 1 day, or few days at the best) of an individual’s SJL status, they are not informative on the long-term effects of circadian misalignment [54]. Again, animal studies may help to fill this strong need for new biomarkers to quantitatively assess the level of SJL, as well as for molecular markers (e.g. proteomics, epigenetics, miRNAs) that are predictive for the onset of chronic disease in later stages of life. Subsequently, these markers have to be validated in human cohort studies. Moreover, such biomarkers would be extremely helpful to determine whether social jetlag should be quantified on the basis of SJL or SJL_SC_ [57,92].

Last but not least, given the high incidence of SJL, even a relatively small increase in health risk will have a significant impact on public health. Therefore, both social and individual strategies should be applied to counteract the adverse effects of SJL. For example, countries should adopt standard time over daylight saving time to counteract partially the incidence of SJL. Additionally, flexible school/work schedules could be a way to decrease the hours of SJL as was demonstrated by the recent studies that surveyed people during the COVID-19 pandemic [66,67,68]. The social restriction in force during the COVID-19 lock-down contributed to minimizing the divergence between the sleep/activity schedules imposed by social obligations and those promoted by the body’s circadian clock. Individual strategies may also be useful. For example, increasing the exposure to blue light in the morning and decreasing it in the evening could help shifting the circadian clock to an earlier phase of entrainment to better adjust to social schedules [69]. The application of social and individual strategies to counteract the effects SJL could improve quality of life in the future.

## Data Availability

Not applicable.
